# Mesenchymal Stem Cells Derived from Human Periapical Cysts and Their Implications in Regenerative Medicine

**DOI:** 10.3390/biomedicines11092436

**Published:** 2023-08-31

**Authors:** Alexandra Roi, Ciprian Roi, Meda Lavinia Negruțiu, Laura Cristina Rusu, Mircea Riviș

**Affiliations:** 1 Department of Oral Pathology, “Victor Babeș” University of Medicine and Pharmacy, Eftimie Murgu Sq. No. 2, 300041 Timișoara, Romania; alexandra.moga@umft.ro (A.R.); laura.rusu@umft.ro (L.C.R.); 2 Multidisciplinary Center for Research, Evaluation, Diagnosis and Therapies in Oral Medicine, “Victor Babeș” University of Medicine and Pharmacy, Eftimie Murgu Sq. No. 2, 300041 Timișoara, Romania; rivis.mircea@umft.ro; 3 Department of Anesthesiology and Oral Surgery, “Victor Babeș” University of Medicine and Pharmacy, Eftimie Murgu Sq. No. 2, 300041 Timișoara, Romania; 4 Department of Prostheses Technology and Dental Materials, Faculty of Dental Medicine, “Victor Babes” University of Medicine and Pharmacy Timisoara, Eftimie Murgu Sq. No. 2, 300041 Timișoara, Romania; negrutiu.meda@umft.ro; 5 Research Center in Dental Medicine Using Conventional and Alternative Technologies, “Victor Babes” University of Medicine and Pharmacy Timisoara, Eftimie Murgu Sq. No. 2, 300041 Timișoara, Romania

**Keywords:** dental mesenchymal stem cells, hPCy-MSCs, tissue engineering, regenerative dentistry

## Abstract

Mesenchymal stem cells currently play an important role in the tissue engineering field in developing new regenerative approaches. The oral cavity is a rich source of mesenchymal stem cells, and introducing the use of dental stem cells, characterized by a multilineage differentiation potential, immunomodulatory activity and repair capacity, offers a good perspective for clinical dentistry. Human periapical cyst mesenchymal stem cells (hPCy-MSCs) represent a new category of dental stem cells, being collected from pathological tissue and exhibiting MSCs-like properties. As studies have described, these new identified cells possess the same characteristics as those described in MSCs, exhibiting plasticity, a high proliferation rate and the potential to differentiate into osteogenic, adipogenic and neural lineages. Reusing the biological tissue that is considered pathologic offers a new perspective for the development of further clinical applications. The identification and characterization of MSCs in the human periapical cysts allows for a better understanding of the molecular interactions, the potential healing capacity and the mechanisms of inducing the local osteogenic process, integrated in the microenvironment. Although their involvement in regenerative medicine research is recent, they exhibit important properties that refer them for the development of clinical applications in dentistry.

## 1. Introduction

Mesenchymal stem cells (MSCs) represent the future in regenerative medicine, offering wide perspectives for clinical applications. One of their main properties is the ability to differentiate into various types of cells, as well as their capacity to self-renew, in order to provide a regenerative action. 

Their initial isolation was performed from the human bone marrow (BMMSCs), requiring invasive surgical procedures and limiting the final number of cells [1]. Currently, alternative approaches for eligible new sources of MSCs are those isolated from blood, adipose tissue, umbilical cord tissue, placenta, muscles, heart tissue and the dental pulp [2,3,4]. Human dental pulp stem cells have exhibited a viable option to be harvested in good conditions, having similar biological properties to the bone-marrow-derived MSCs [5]. There are studies that discuss and outline the advantages of using MSCs through their potential of forming high cell colonies, with a higher proliferation and survival rate compared to bone-marrow-derived MSCs [6]. 

Mesenchymal stem cells are described as multipotent cells with the property of differentiating into various cells lineages, possessing a high capacity for self-renewal and proliferation [7]. These capacities have been shown in multiple in vitro studies, which have achieved their differentiation into fibroblasts, adipocytes, chondrocytes, myocytes, pancreatic cells and hepatoblasts [8,9]. As these cells are defined by being important mediators of the immune system response, their main action focuses on a high implication of tissue repair and suppressing chronic inflammation in the surrounding environments by exhibiting a regenerative function.

Regenerative medicine and tissue engineering focus on implementing the potential of MSCs by improving the interaction between cells and proteins, in order to generate new tissue cells. In the study conducted by Rochira et al. [10], they outline the importance of obtaining a higher regenerative bone process through cell-based therapy. Focusing on this desiderate, they revealed the effect of the concentrated growth factor (CFG) upon the human bone marrow stem cells (hBMSCs) in an in vitro study. Their results show that the hBMSCs exhibited a higher osteogenic differentiation potential under these conditions of incubation. One of the main identified advantages of MSCs is their high potential to generate osteoblastic transformation, with important implications in future bone-guided regeneration [11]. The study conducted by Palermo et al. [12] aimed to activate the osteogenesis process and stimulate the differentiation of hMSCs while using dental implants coated with CFG. The results showed an improved adhesion of the endothelial cells to these implants, compared to the control group represented by implants without CFG coating. Also, the introduction of different types of scaffolds can successfully influence the guided bone regeneration process, as Giannotti et al. [13] revealed in their study by using hydroxyapatite- silicon scaffolds to promote the osteogenic differentiation process.

Dental-derived stem cells (DSCs) have a wide applicability and can provide a broad range of therapeutic approaches, as research has revealed the potential transformation of dental stem cells into chondrocytes [14] and into cardiomyocytes [9], this being an important step towards the regenerative cell-based medicine.

The accessibility of dental stem cells has caused a rise in interest among researchers, due to the easy and less invasive isolation technique compared to the BMMSCs. There are studies that have outlined their osteogenic potential in both in vitro and in vivo cases, providing results that discuss the existing phenotypic differences among the different types of dental MSCs, with various potential applications [15]. The dental-derived stem cells originate from different tissues of the oral cavity, such as the gingival tissue [16,17], periodontal ligament [18], dental pulp [19], dental follicle [20] and the apical papilla [21]. 

There are several studies that discuss the potential of the dental pulp stem cells (DPSCs), and of the stem cells that are derived from the exfoliated deciduous teeth, to further differentiate into neurons [22]. Arthur et al. [23], in their in vivo study, showed the active differentiation of the DPSCs into neuronal cells in specific environment conditions. Nevertheless, dental-derived stem cells are a viable option under certain conditions, taking into consideration reports that outline their higher number in younger subjects, as well as their presence in case of third molar dental follicles. 

Studies have shown an important aspect related to the potential use of dental mesenchymal stem cells derived from inflamed periodontal ligament cells and inflamed periapical tissue [24,25]. These types of stem cells are easy to harvest and have exhibited numerous properties related to their high differentiation and regeneration rate, similar to those of the MSCs obtained from healthy tissues. It appears that the MSCs derived from pathological tissues such as periapical lesions possess a particular immunophenotype with a strong ability to differentiate into osteogenic cells [26]. 

Human stem cells offer a wide perspective in developing new regenerative approaches, focusing on the implementation of easy, simple and effective techniques that can offer more applications. The dental-derived stem cell population, when compared to the existing “gold standard” represented by the bone-marrow-derived mesenchymal stem cells, exhibited important properties relating to their differentiation potential, being able to give rise to at least three cell lineages, with important implications in the odontogenic and osteogenic populations [21]. An important aspect that has been discussed is the implication of the mesenchymal stem cells derived from periapical lesions and their osteogenic potential. Taking into consideration the isolation of these types of cells in the periapical pathological tissue, their isolation and use in regenerative dentistry offers important advantages in the future of the management of the cases. 

The aim of the present review is to outline the potential for the use of dental-derived mesenchymal stem cells in regenerative dentistry, focusing on the implications of the MSCs isolated from periapical pathological lesions. Knowing the implications of these cells in tissue engineering, by reviewing the existing literature data, this review highlights their characteristics and potential clinical implications and applications as future regenerative alternatives in the dental field.

### 1.1. Biological Properties of Mesenchymal Stem Cells

Alexander Friedenstein was the one who first described the existence of a non-hematopoietic cell population that was able to differentiate and renew, being localized in the bone marrow [27]. Further researchers have proved that the cell population that was described by Friedenstein, while isolated through his technique, had a high proliferation rate and the potential to differentiate into different mesenchymal tissues; therefore, their name became “mesenchymal stem cells” [28]. It appears that not all the cells from this category possess the same stem cell criteria, with variations in the differentiation potential, phenotype and gene expression [29]. 

**Figure 1:**
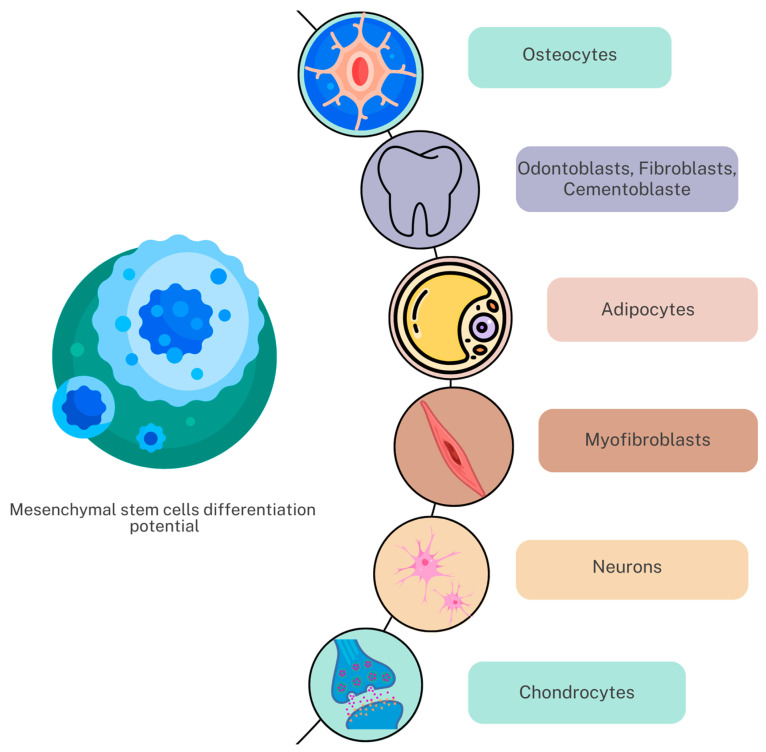
Mesenchymal stem cells’ differentiation potential.

Currently, a cell population that has the following characteristics, with no consideration of its source, is considered to be in the “mesenchymal stem cell” category: having specific morphology and properties to adhere to plastic, with fibroblast-like characteristics, and having a high self-renew rate and a possibility of differentiating into various mesenchymal lineages (such as chondrocytes, adipocyte or osteocyte) (Figure 1). Research has outlined how wide in vitro potential in human mesenchymal stem cells from multiple sources can differentiate into ectoderm- and endoderm-derived lineages, such as hepatocytes and neurons [30], under specific growth conditions. 

Studies have shown that the mesenchymal stem cell population under specific conditions of stimulation can produce multiple differentiated cell types. Their division capacity is controlled by environmental and developmental signals that control the adequate number of the stem cells, as well of the resulting differentiated cells [31]. Nevertheless, the differentiated cells have a limited division capacity, due to the consequence of losing their telomere length after each proliferative step. As for the MSCs, research has revealed that they are able to proliferate in vitro into more than ten passages, without interfering with their original characteristics [32]. 

Among their abilities to repair or replace affected tissues, MSCs exhibit another important action that modulates the immune system response. They are capable of interacting with different categories of immune cells (natural killer, T lymphocytes, B lymphocytes and dendritic cells) and have an anti-inflammatory action by inducing the apoptosis and limiting the proliferation of the immune cells [33]. 

These important characteristics and biological implications of MSCs are an asset in tissue engineering and regenerative medicine that can improve the approaches and the outcome, offering a wide range of applications.

### 1.2. Dental Mesenchymal Stem Cell Sources

Research has shown that mesenchymal stem cells can derive from orofacial tissues, and represent an important postnatal stem cell source, especially for hard tissue regeneration. Nevertheless, it was reported that this category of stem cells has superior osteogenic regeneration potential compared to the BMMSCs [32]. Taking into consideration that the orofacial tissue derives from the neural crest, dental mesenchymal stem cells have a greater potential to differentiate into neural crest-derived oral tissues [34]. 

The dental tissue represents a specialized tissue that, compared to the bone one, does not undergo a continuous remodeling process. Over the years, the dental stem cell population has been isolated and studied, showing a wide range of characteristics. The first category identified and described was the DPSCs [35], which have the capacity to differentiate into multiple lineages, with an important involvement in further odontogenic development, rather than osteogenic, forming a dentin-like tissue [36]. Research revealed the fact that the DPSCs exhibited higher in vitro proliferation activity compared to that of BMMSCs, with various differentiation patterns, mainly similar to that of the microenvironment they derived from [37]. 

**Figure 2:**
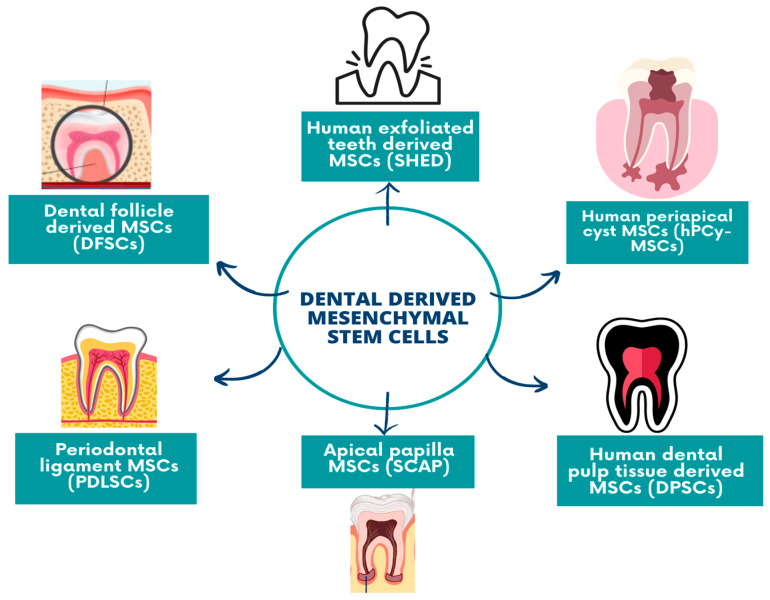
Dental-derived mesenchymal stem cells.

Another source of dental mesenchymal stem-cells studied was their isolation from human exfoliated deciduous teeth (SHED), as this type of MSCs could differentiate into multiple lineages such as chondrogenic, adipogenic, or osteogenic [38,39]. Compared to the DPSCs and BMMSCs, SHED had a higher proliferation rate and differentiation potential. In the dental field, as research progressed, more mesenchymal stem cell sources were described and further isolated: apical papilla (SCAP), dental follicle precursor cells, MSCs from the gingiva, periodontal ligament derived stem cells (PDLSCs), orofacial bone-marrow-derived stem cells (OMSCs) and, more recently discovered, the periapical cyst-derived mesenchymal stem cells (hPCy-MSCs) [40,41,42,43] (Figure 2).

The implications of dental stem cells in regenerative medicine focus on their ability to limit the inflammatory state and promote migration and proliferation, preventing apoptosis and inducing the tissue repair process [44]. All these actions are possible through their immunomodulatory action due to their secretome [45]. In this case, the periapical inflammatory tissues that form as a response to endodontic infections can actually express MSC markers and may contain osteogenic precursors that have the potential to differentiate into mature osteoblastic cells, with an implication in the local regenerative process [46]. A study conducted on an animal model identified that MSCs were present in the granulation tissue that formed as a response to a local action [46].

Given the high accessibility, easy harvest and important potentials of the different types of dental mesenchymal stem cells, regenerative medicine is entering into a new era. Taking into consideration the existing evidence on the presence of MSCs in human periapical cysts, a new perspective is given for the regenerative approaches. 

## 2. Mesenchymal Stem Cells in Human Periapical Cysts

To date, as research has revealed, there have been several types of dental stem cells isolated from teeth (stem cells from the dental pulp, from human exfoliated teeth, periodontal ligament stem cells, stem cell from apical papilla and those from dental follicles). It was recently reported that mesenchymal stem cells were successfully isolated from inflamed periodontal ligaments [25] and human pulp tissue [47], providing a new perspective in the use of MSCs from pathological dental tissue, as they seemed to have the same characteristics as those from the healthy tissue [24]. 

Human periapical cysts represent an inflammatory response to an endodontic infection and are considered to be the progression of chronic apical periodontitis. Studies mention the fact that a chronic apical periodontitis will not evolve towards the development of a periapical cyst in all cases. Maeda et al. [47] reported that in the case of some periapical lesions, they could identify osteogenic cells that would differentiate into osteoblastic ones. In this case, surrounding bone healing is stimulated. The study performed by Patel et al. [48] on an animal model showed the fact that mesenchymal stem cells were present in the periapical granulation tissue that developed as a consequence of the response to the identified foreign bacteria. In the study conducted by Liao et al. [24], they identified the presence of MSC markers in the inflammatory human periapical tissue, a fact that implies the presence of MSCs, as well. Nevertheless, in this study they managed to isolate the cells and identify the mesenchymal cell phenotype with the potential to differentiate into osteocytes and adipocytes. 

In 2013, Marelli et al. [46] were the first to isolate and describe the existence of mesenchymal stem cells in human periapical cysts, using the term human periapical cyst-mesenchymal stem cells. They identified these mesenchymal stem cells in the inner layer of the periapical cyst’s wall, successfully isolated and exhibiting specific characteristics that could separate them from the present pathological tissue. Further studies have shown that the hPCy-MSCs had similar features to the other categories of MSCs, showing a high affinity towards differentiation into osteogenic lineages. The surface cell markers, especially the CD146+ expression, influence the further behavior of the mesenchymal cells. It appears that a lower expression of CD146+ is correlated with an increased proliferation rate and a higher osteogenic activity [49] (Table 1). Further, it appears that hPCy-MSCs in certain conditions and under neurogenic stimulating environments express high levels of neuronal markers, differentiating into dopaminergic neurons [50]. In the same study, the authors outlined the differentiation potential of this category of cells towards the adipose lineage. In order to evaluate this property, specific staining was used upon the incubated cultures and the presence of triglyceride deposits was revealed. To sustain these findings, while evaluating the gene expressions of several specific adipogenic markers, the results showed increased expressions of lipoprotein lipase, glucose transporter type 4, adiponectin and peroxisome proliferator-activated receptor gamma. In the research conducted by Liao et al. [24], which aimed to evaluate the in vitro differentiation potential of the MSCs encountered in the periapical tissue, their results described a mild differentiation capacity into adipocyte-like cells.

Also, Marelli et al. [50] conducted another study, aiming to identify the differentiation potential towards the neurogenic lineage of hPCy-MSCs and their possible involvement in the treatment of neurodegenerative diseases. Their results revealed a high neurogenic potential, similar to that of the DPSCs, with a possible application in the future treatment of cell therapies. 

The results of the study performed by Dokic et al. [26] in which they evaluated the immunomodulatory and anti-inflammatory action of the MSCs, show the fact that the MSCs do not determine the proliferation of the mononuclear cells, providing an immunosuppressive action. These aspects suggest that the characteristics of MSCs are a useful tool for limiting the chronic inflammatory process and induce tissue repair by promoting osteoblastic proliferation. 

In the study led by Araujo et al. [51], the authors aimed to identify the participation of MSCs in the periapical granuloma development. Their results showed the presence of multiple MSC markers in inactive periapical lesions, compared to the active ones. Also, it appears that the presence of the MSCs has a strong relationship with the immunosuppression present in the local environment, suggesting that their role could be in minimizing the extent of the periapical inflammatory lesions while inducing the tissue repair process (Table 1). 

A new perspective was presented by Chrepa et al. [52], in their study, by collecting blood samples from teeth with intracanal bleeding evoked from the periapical lesions, followed by the isolation of the identified MSCs in these samples. The intracanal MSCs were cultured in osteogenesis basal media for two weeks, and afterwards their osteogenic potential was quantified. A comparison between the levels of MSC markers present in obtained systemic blood samples and the samples from the bleeding root canals revealed upregulated levels of the positive markers CD90, CD73, CD105 and CD146, while downregulated levels were identified in case of the negative marker CD45. These findings are according to the existing literature data, which characterizes the identification of mesenchymal stem-like cells through these altered marker expressions [53]. Also, while isolating an MSC population from the periapical lesion, these cells formed a colony, adhered to plastic and differentiated into a mineralized phenotype while exposed to a proper in vitro environment. The periapical lesions underwent histopathological examination and immunohistochemical staining, the results identifying the same surface markers. These findings suggest the possibility that an over-instrumentation of the canal transferred the MSCs from the periapical lesion into the canal. Given this active transfer, the possibility of using the presence of MSCs in the chronic periapical lesions could be an attempt at the regenerative procedure involving the pulp–dentin complex. 

Estrela et al. [54] discussed the presence of MSC in chronic periapical lesions and primary periapical lesions. The presence of the MSCs in periapical lesions has been intensively discussed, with the fact that they could contribute to the development of an immunosuppressive local environment that limits and stabilizes the periapical lesions being mentioned. The results of the study revealed that in case of the persistent chronic periapical lesions, the marker expression of CD90 was higher and the MSCs identified could contribute to the maintenance of an immunosuppressive and stable microenvironment of the chronic periapical lesion. Another study conducted by the same authors that included samples from periapical cysts, periapical granulomas and periapical abscesses aimed to identify the presence and the role of MSCs [55]. The results showed that the MSCs were present, characterized through the immunohistochemical staining of the surface markers. Parts of the surface markers CD44 and CD73 were positive in the chronic inflammatory infiltrate from the capsular connective tissue in periapical cysts samples. An aspect they encountered was the fact that acute periapical lesions were a rich source of MSCs, a fact that could be explained by the local inflammatory conditions. Although the entire process that acute periapical lesions undergo to transform into periapical cysts is not completely known, studies have proved that the MSCs remain viable in the inflamed tissue, being isolated from the capsule of the periapical cysts, as well [46]. A theory that follows the pathway of the transformation into periapical cysts is based on the fact that the MSCs are activated by the inflammatory conditions and contribute to the pathogenesis of the periapical cysts [55]. One of the main questions remains related to the presence of MSCs in the periapical cysts. Studies discuss the fact that their presence exhibits a strong paracrine effect upon the surrounding cells, with a strong proliferative action that determines the formation of the epithelial lining of the cyst [56]. Nevertheless, research suggests that the presence of the MSCs in periapical cysts has an important osteogenic effect, actively promoting healing [51]. It has been discussed that the source of the MSCs in the periapical lesions could be due to the remnants of odontogenesis or from the stem cells present in the periodontal ligament [46]. Another explanation could be that the Malassez cells are one of the main sources in the periapical area [57]. 

Tatullo et al. [58], in their study, compared the osteogenic potential of hPCy-MSCs and of DPSCs. By cultivating them in an osteogenic environment, the results outlined their potential to differentiate into osteoblastic cells (Table 1). The data obtained after performing the RT-PCR (Real-Time Polymerase Chain Reaction) showed that although their differentiation potential was similar, there were certain differences in the expression of two genes, the Dentin Matrix acid Phosphoprotein (DMP-1) and Dentin Sialophosphoprotein (DSPP). These two genes exhibited an increased expression in the case of the differentiated DPSCs in comparison to the results seen in the case of the hPCy-MSCs. In order to outline the implications in the osteogenesis process and odontogenic differentiation, the presence of DMP1, DSPP, OPN (osteopontin) and OSC (osteocalcin) are considered markers that are present in the dentin, cementum and bone [49]. Also, related to the hPCy-MSCs lineage, Runt-related transcription factor 2 (RUNX-2), Osteonectin (ON) and Bone sialoprotein (BSP) showed an increased expression in the case of hPCY-MSCs, compared to the DPSCs. The results outline that the differentiation potential of hPCy-MSCs is predominantly towards bone regeneration, and afterwards they also exhibited markers involved in the dentinogenesis process, while the DPSCs exhibited a clear evolution and implication in the odontogenesis process. The differentiation potential of hPCy-MSCs into adipose lineage was outlined by Marelli et al. [46], in their study, showing an increased expression of adipose-specific genes while performing the RT-PCR analysis. 

Human periapical cyst mesenchymal stem cells have been reported to have a high proliferation rate and large differentiation options. One of the main properties of hPCy-MSCs is their involvement in the bone regeneration process. In order to outline this potential, hPCy-MSC have been added to specific scaffolds, such as synthetic poly lactic-co-glycolic acid (PLGA) and chitosan scaffolds, exhibiting excellent chemical and physical properties for bone regeneration approaches [59,60,61]. Also, another appealing approach in the research field is represented by the affinity of the hPCy-MSCs towards the development of neuron-like cells and their use in degenerative disorders [62,63]. Li et al. [64] conducted a study aiming to compare the properties of the periapical lesions derived from MSCs and from the DPSCs. They evaluated the differentiation potential, the immunomodulatory properties and the pro-angiogenesis ability. The results show that the proliferation and migration abilities were similar between the two categories of MSCs. As for the osteogenic differentiation potential, the periapical-lesion-derived MSCs were able to induce an osteogenic process, with the formation of calcified nodules. The pro-angiogenesis ability was more representative in the case of the periapical-lesion-derived MSCs, suggesting a more effective approach in the initialization of the angiogenesis process. Immunomodulatory activity was evaluated by assessing the levels of the immunomodulatory genes, and they had an increased expression in the case of the periapical lesion MSCs. 

**Table 1:** Differentiation potential of hPCy-MSCs.

Reference	Type of Study	Type of Samples	Identified Potential hPCY-MSCs	Differentiation Potential
[47]	In vitro	Periapical chronic granulation tissue	MSC-like cells	Osteogenic differentiation and production of calcified deposits
[24]	In vitro	Human periapical inflammatory tissue	MSC-like cells	Osteogenic differentiation potential
[48]	In vivo	Cell transplantation in mouse models	MSCs-like cells	Osteogenic differentiation and formation of mineralized tissue
[46]	In vitro	Human periapical cystic tissue	MSC-like cells	Osteogenic and adipogenic differentiation
[49]	In vitro	Human periapical cystic tissue	MSC-like cells	High osteogenic differentiation potential
[50]	In vitro	Human periapical cyst tissue	MSC-like cells	Neurogenic differentiation potential
[26]	In vitro	Human periapical lesions tissue	MSC-like cells	Osteogenic, adipogenic and chondrogenic differentiation potential
[51]	In vitro	Human periapical granuloma tissue	MSC-like cells	Immunomodulatory action and local healing
[54]	In vitro	Human periapical lesions	MSCs-like cells in inflammation infiltrate	Immunomodulatory response and pro-healing action associated with primary apical lesions
[55]	In vitro	Human periapical lesions	MSCs-like cells from the periapical lesions	Immunomodulatory action; a higher quantity of cells in acute lesions, suggesting their implication in the progress of the lesion
[58]	In vitro	Human periapical cysts	MSCs-like cells from periapical cysts	Osteogenic differentiation potential with an increased expression of the specific genes.
[64]	In vitro	Human periapical lesions	MSC-like cells	Osteogenic differentiation Immunomodulatory abilities Pro-angiogenetic activity

The presence of mesenchymal stem cells in periapical cysts outlines the new perspectives related to the management of the cases. Although their osteogenic differentiation potential has been reported multiple times, their local immunomodulatory action is an important aspect that is another characteristic of these cells. Studies have reported that the hPCy-MSCs were able to reduce the periapical inflammatory cells, controlling the peripheral blood mononuclear cells and reducing the local tissue injury in this manner [63]. Their use for tissue engineering is an important aspect that needs to involve the quantification of all the properties that these cells have in developing multiple cell lineages. 

## 3. Clinical Applications of hPCy-MSCs and Future Perspectives

In the past years, research has proved the high potential of stem cells in tissue engineering and their implications in regenerative medicine. Given the important properties of stem cells, related to their self-renewal capacity and high differentiation potential, they have become one of the main topics for clinical applications [65]. Although adults have multiple stem cells sources, the harvesting of dental mesenchymal stem cells is a non-invasive method, and less painful, especially considering that the hPCy-MSCs are considered pathological tissue, a waste with no further clinical implications. As reported, dental mesenchymal stem cells appear to have the regenerative potential of the environment in which they were harvested. As a consequence, clinical applications need to take into consideration the location-associated characteristics, it being revealed that periodontal ligament-derived stem cells have a higher differentiation rate into cementoblasts [66] and collages fibers that assemble the Sharpey ones [67]. Dental-pulp-derived stem cells have shown a higher ability to produce similar dentine-specific components [68], while several studies have reported that dental MSCs were able to induce osteogenesis in animal calvaria bone defect models [69]. Neurogenic differentiation was also achieved by using odontogenic MSCs that transformed into neuron-like cells [70], with the possibility of being introduced in the treatment of neurodegenerative diseases. These cells also showed potential to be used in the treatment of ischemic vascular [71] and liver diseases [72]. Yokoyama et al. [72], in their study, showed the potential of exfoliated deciduous tooth stem cells to repair the liver structure and limit liver fibrosis in rat models. 

The ability of dental mesenchymal stem cells to secrete specific components such as cytokines, growth factors, antioxidants and angiogenic factors outlines their ability to be used based on their immunomodulatory functions in the treatment of autoimmune diseases [73]. Based on their secretome properties, the MSCs promote a decrease in inflammation, and the migration, proliferation and differentiation phases specific for a regenerative action. For tissue engineering and in order to provide the desired regenerative potential, stem cells should fulfill several criteria [74], such as having a high number and multilineage differential potential, a minimally invasive approach for their collection, following the guidelines of the good manufacture practice and having the possibility to be safely transplanted to a host (autologous or allogeneic). 

The hPCy-MSCs category is the most recently discovered, and taking into consideration that their origin is in pathological tissue, this makes them appealing for further research. Based on the theory that MSCs exhibit higher differentiation potential into the tissue cells from where they are harvested, studies have shown that when they are added to scaffolds, they have the ability to form bone tissue [75]. Based on their regeneration and differentiation potential in various conditions, hPCy-MSCs can develop into neural phenotypes and could be used in cell-based therapy approaches for the management of Parkinson’s and Alzheimer’s disease [50]. Also, based on their characteristics and interactions, they have great potential to be used in maxilla facial bone regeneration. Taking into consideration that the process of bone regeneration includes the involvement of both differentiated osteoblasts and the angiogenesis process, the use of local MSCs in their local environment with these specific abilities could be the answer [76,77]. 

Dental periapical cysts are among the most common pathologies encountered in the dental field, being characterized by chronic evolution and needing a surgical treatment approach. Based on the presence of MSCs markers in the pathological tissue, these cells were isolated from the wall lining of the cysts, and their differential potential and characteristics were evaluated. The existence of hPCy-MSCs opens up new perspectives in the management of bone regeneration, and tissue engineering can develop new strategies for the use of so called “biological waste” tissue. Currently, the challenge consists of translating the in vitro results into in vivo clinical outcomes, as there are concerns related to the use of periapical pathological tissue. Being limited to in vitro studies, with cell cultures being treated under a specific protocol, the clinical limitations when introduced as an in vivo approach are not yet known. Although Marelli et al. [46], when they characterized this population, could successfully separate them from the pathological periapical tissue, and Araujo et al. [51] described their presence in the inactive lesions, discussing their immunomodulatory actions and involvement in the tissue repair process, concerns regarding the biological safety should be addressed in future research. 

## 4. Conclusions

The use of dental-derived MSCs has multiple advantages, starting from their easy harvest and ending with their important regenerative and immunomodulatory interactions. These MSCs possess a multilineage differentiation potential, and research has outlined their capacity to develop into functional cells. In the present review, we focused on the implications of hPCy-MSCs and their regenerative potential in dentistry. As studies have shown their ability to develop into osteoblasts, neurogenic-like cells and adipocyte-like cells, their immunomodulatory action still needs to be evaluated in vivo. By introducing this new category of dental stem cells, regenerative medicine and tissue engineering can be introduced to new approaches using these cells in dentistry.
